# An Assessment of Surface Contamination and Dermal Exposure to 5-Fluorouracil in Healthcare Settings by UPLC-MS/MS Using a New Atmospheric Pressure Ionization Source

**DOI:** 10.3390/toxics12110766

**Published:** 2024-10-22

**Authors:** Matteo Creta, Eline Verscheure, Birgit Tans, Herman Devriese, An Devriendt, David Devolder, Robin Lebegge, Katrien Poels, Lode Godderis, Radu-Corneliu Duca, Jeroen A. J. Vanoirbeek

**Affiliations:** 1 Centre for Environment and Health, Department of Public Health and Primary Care, University of Leuven (KU Leuven), 3000 Leuven, Belgium; matteo.creta@lns.etat.lu (M.C.); eline.verscheure@kuleuven.be (E.V.); robin.lebegge@kuleuven.be (R.L.); katrien.poels@kuleuven.be (K.P.); lode.godderis@kuleuven.be (L.G.); radu.duca@lns.etat.lu (R.-C.D.); 2 Unit Environmental Hygiene and Human Biological Monitoring, Laboratoire National de Santé (LNS), Department Health Protection, 3555 Dudelange, Luxembourg; 3 Hospital Pharmacy UZ Leuven, University Hospital Leuven (UZ Leuven), 3000 Leuven, Belgium; birgit.tans@uzleuven.be (B.T.); david.devolder@uzleuven.be (D.D.); 4 Safety—Health and Environment, University Hospital Leuven (UZ Leuven), 3000 Leuven, Belgium; herman.deviese@uzleuven.be; 5 IDEWE, External Service for Prevention and Protection at Work, 3001 Heverlee, Belgium; an.devriendt@idewe.be

**Keywords:** 5-fluorouracil, antineoplastic drug, occupational exposure, occupational exposure

## Abstract

5-Fluorouracil (5-FU) is a well-known cytostatic drug, which is often used in cancer treatments. Yet, it is also a very dangerous compound for people who are occupationally exposed to it for a long time, such as pharmacy employees, nurses and cleaning staff. We aimed to improve and implement a LC-MS/MS method for 5-FU quantification on surface contamination samples collected with swabs in a pharmacy department and outpatient nursing station of a university hospital. To improve the existing methods to quantify 5-FU, we compared a LC-MS/MS method using the frequently applied electrospray ionization source (ESI) with a UniSpray ionization source (USI). To determine the contamination of 5-FU in a pharmacy department preparing 5-FU infusion bags, which are then given to patients in the outpatient nursing stations, we collected multiple surface swabs of the laminar flow cabinets and frequently touched objects, before the preparation and administration of 5-FU and afterwards. Furthermore, we sampled the protective gloves and the bare hands of employees of the pharmacy department, involved in the preparation of the infusion bags. Using the USI source, we were able to reach the lowest limit of quantification (LOQ). With this technique, we were able to detect 5-FU contamination on the laminar flow cabinets and frequently used objects in the pharmacy department and the outpatient nursing station in the very low ng/cm^2^ range. This contamination was mostly higher after preparation or administration than before. While we also found 5-FU on the protective gloves, we almost found no 5-FU on the skin of the pharmacy technicians preparing the 5-FU infusion bags. In conclusion, our method was able to detect very low concentrations of 5-FU contamination, but the contamination we found is very unlikely to result in any issues for the personnel working in these areas.

## 1. Introduction

5-Fluorouracil (5-FU) is a synthesized anticancer drug developed in the previous century and extensively used to treat multiple types of cancer, including esophagus, stomach, pancreas, and colorectal cancer [1,2,3]. Since 5-FU is poorly absorbed after oral administration, intravenous administration as bolus or continuous infusion is mostly used [4]. Internally, 5-FU is converted from the parent compound to an active metabolite that disrupts both DNA and RNA synthesis, exerting its anticancer activity in this way [5]. The interaction with DNA is not specific, nor selective. Therefore, exposure to 5-FU is also known to induce organ damage, infertility, etc., in healthy persons. Hospital personnel, such as doctors, nurses, pharmacists or auxiliary personnel, manipulating or accidently coming into contact with cytostatic drugs, may be exposed to this hazardous compound [6,7].

In recent decades, several actions were taken to minimize exposure to anticancer drugs, such as using an efficient laminar flow cabinet for the preparation of 5-FU infusion bags, increasing the protection of the workers, using closed system drug-transfer devices, or improving the cleaning efficiency [8,9,10,11]. Specific occupational exposure limits (OELs) or threshold limit values (TLVs) are not yet established for anticancer drugs, yet several recommendations and guidelines are published [12,13]. These actions are clearly an essential step forward in reducing occupational exposure to 5-FU. Nevertheless, measuring workplace exposure is still needed to verify if the precautions taken are effective. Different analytical and sampling methods are available to monitor exposure to 5-FU via surface contamination and dermal exposure. Due to the non-volatility of 5-FU, inhalation exposure is considered less important [14].

To analyze 5-FU samples, mostly liquid chromatographic methods are used, due to the high sensitivity and the ability to detect low concentrations [15,16,17,18]. Lately, liquid chromatography (LC) is coupled by an atmospheric pressure ionization (API) source to a tandem mass spectrometric (MS/MS) detection, to further increase the sensitivity and specificity of the analysis [19,20,21]. Such methods, based on the electrospray ionization (ESI) source, are generally able to determine concentrations in the range of ng/cm^2^ [6]. However, due to the high chemical risk profile of this drug, there is a constant interest in developing ultra-sensitive methodologies able to detect extremely low concentrations of 5-FU. The aim of this study was to evaluate surfaces contamination, and possible dermal exposure, for pharmacy personnel involved in the preparation of 5-FU, through the development of a very sensitive Ultra-Performance (UP)LC-MS/MS technique.

## 2. Materials and Methods

### 2.1. Chemicals and Materials

LC–MS-grade acetonitrile (CH_3_CN), methanol (MeOH) and water were obtained from Biosolve (Valkenswaard, The Netherlands). 5-Fluorouracil (5-FU) and 5-Fluorouracil 13C 15N (99.8%), formic acid (CH_2_O_2_), ammonium hydroxide (NH_4_OH) (28%), ammonium formate (NH_4_HCO_2_), and glass vials (20 mL, thread 18, O.D. × H 22.5 mm × 75.5 mm), and with screw top were obtained from Sigma-Aldrich (Saint Louis, MO, USA). The sampling swabs TX 714K were purchased from Texwipe (Kernersville, NC, USA); an automatic shaker (Heidolph Scientific Products GmbH, Schwabach, Germany)) was used during the sample preparations.

### 2.2. Preparation of Stock and Working Solutions

A stock solution of 1 mg/mL 5-FU was made by weighing 10 mg of 5-FU and diluting with 10 mL of acetonitrile. A stock solution of 5-FU 13C 15N, used as internal standard, was made by mixing 1.73 mg with 10 mL of acetonitrile. Both stock solutions were stored at −20 °C. The stock solution of internal standard was progressively diluted with acetonitrile to obtain an extraction solution with a final concentration of 1 ng/mL of 5-FU 13C 15N. The 5-FU stock solution (173 µg/mL) was then progressively diluted with MeOH/H20 (8/2) to obtain a 10 mL aliquot of the following calibration working solutions: 0.5, 1, 3, 7, 10, 70, 100, 300, 500, 700, 1000 ng/mL.

### 2.3. Extraction Efficiency and Linearity Testing

A recovery efficiency test was performed to determine whether the 5-FU could be recovered from the Texwipe swab using acetonitrile as extraction solvent. In this test, known amounts of 5-FU (0.5, 1, 10, 50, 100 ng/swab) were spiked on the sampling material of the swabs. After this, the samples were treated following the same procedure described in the sample preparation paragraph and injected on the UPLC-MS/MS instrument. The extraction efficiency was calculated dividing the relative peak area of the spiked swabs by the relative peak area of standard solutions of 5-FU. This test was performed in pentaplicate.

To determine the linearity, we spiked 0.1 mL of the calibration solution (internal standard and extraction solution) on Texwipe sampling swabs, to obtain the following amounts of 5-FU on the sampling material: 0.05, 0.1, 0.3, 0.7, 1, 7, 10, 30, 50, 70, 100 ng/swab (in triplicate). Again, a similar sample treatment and UPLC-MS/MS analysis was performed.

### 2.4. Sample Preparation

The spiked Texwipe swabs used for the calibration, and the collected samples, were extracted in a 20 mL glass vial using 10 mL of an acetonitrile solution containing 1 ng/mL of internal standard. The samples were then shaken for 30 min using an automatic shaker. From this solution, 9 mL was transferred to a 10 mL glass tube and dried out under a gentle nitrogen stream at 40 °C. Next, the sample was reconstituted with 300 µL of acetonitrile in a high-recovery glass vial. From this solution, 10 µL was injected in the UPLC-MS/MS instrument.

### 2.5. UPLC-MS/MS

The analyses were performed using a UPLC system consisting of a Waters Acquity H-Class UPLC coupled with a Waters Acquity H-Class Sample Manager FTN (Waters, Manchester, UK). We used an Acquity UPLC BEH Amide, 2.1 × 50 mm, 1.7 µm column, with the thermostat at 40 °C. The mobile phase solvent A consisted of H_2_O with ammonium formate (10 mM) and ammonium hydroxide (NH_4_OH) (0.05%); the mobile phase B consisted of acetonitrile with 0.01% of formic acid. The initial mobile phase composition was 95% solvent B and 5% solvent A, pumped at a flow rate of 0.4 mL/min for 0.3 min. From 0.3 to 1.2 min, solvent A was increased linearly from 5 to 60%. These settings were held for 0.6 min. From 1.8 to 2.3 min, solvent B was decreased to 5% and kept for 0.7 min, after which the next sample was injected. The overall run time was 3 min.

The mass spectrometric detection was carried out using a Xevo TQ-XS Tandem Quadrupole Mass Spectrometer (Waters, Milford, MA, USA). We tested two different types of ionization source, namely (1) the more classical electrospray ionization source (ESI) and (2) the UniSpray ionization source (USI). In both cases, the negative-ion and multiple reaction monitoring (MRM) modes have been used. The comparison between ESI and USI involved the determination of parameters such as limit of detection (LOD) and limit of quantification (LOQ), and the linearity and signal-to-noise (S/N) ratio, after injecting a calibration curve, performed in triplicate.

The settings of the mass spectrometer for USI and ESI are shown in Table 1. In both cases, quadrupoles 1 and 2 were set at 2.8 for low mass resolution and 15 for high mass resolution. The dwell time was 0.03 s, and the interscan delay was 0.2 s. The span was set at 0 a.m.u. The LC system and mass spectrometer were controlled by Waters MassLynx software (version 4.0), and data were collected with the same software.

### 2.6. Method Validation

After selecting between ESI and USI as the best ionization source, the method was validated for linearity of the calibration curves and the associated coefficient of determination R^2^, limit of quantification (LOQ), intra- and inter-assay accuracy and precision in accordance with international rules [22].

The calibration curves were fitted by linear regression analysis on the peak area ratios of the target compounds to the IS, against the different nominal concentrations of the target compounds. The fitting of the calibration curves was evaluated using the error on the calculated concentrations expressed as percentage of target concentrations (% of target) and the coefficient of determination R^2^ of the calibration curves. According to the above-mentioned international rules, the deviation of the measured concentration from the nominal concentrations should not exceed 10%.

The LOD was determined using a signal-to-noise approach, considering a signal-to-noise ratio of 2:1. The LOQ was defined as the lowest concentration that could be quantified with an acceptable level of accuracy (relative standard error (RSE) ± 20%) and precision (RSD < 10%).

### 2.7. Evaluation of Exposure to 5-FU in a Public Hospital

#### 2.7.1. Surface Sampling

The surface contamination of 5-FU was evaluated in a public hospital in Belgium, where 5-FU was extensively used with an estimated amount of 15 kg manipulated every year. Surface wipe sampling was performed in the following two environments of the hospital: (1) the pharmacy department (PD) where 5-FU was prepared, and (2) the outpatient nursing stations (ONS) where patients were treated with 5-FU.

**Figure 1:**
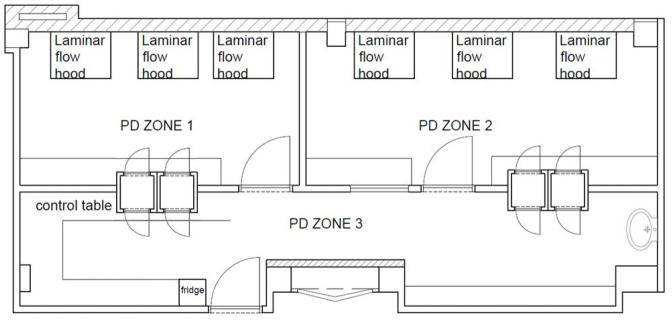
A map of the pharmacy department (PD) of the hospital (compounding zone) where the 5-FU infusion bags were prepared and collected.

Figure 1 shows the PD consisting of the three following compartments: PD zone 1 and PD zone 2 were used for the preparation of the infusion bags under laminar flow cabinets (LFC) (three LFC are present in PD zone 1 and three in PD zone 2), while PD zone 3 was used for the storage of drugs and the collection and final check of the prepared bags. In zone 3, different electronic devices, such as computers, telephones, and fridges, are present. Two surface samples were taken, one before (immediately after cleaning) and one after the preparation of 5-FU infusion bags. The cleaning procedure that is in place depends on the following specific environment: the laminar flow cabinets were cleaned every day by the pharmacy technicians (three times per day: morning, lunch break and evening) using Klercide Neutral Detergent^®^, followed by Ethanol 70%. PD zone 3, and all the devices present in the room, such as the telephones, keyboards, and mice, were cleaned by cleaning staff using strong household cleaning products.

In ONS (administration site), patients were treated with 5-FU in 2 different rooms, ONS1 and ONS2, with a toilet present in each room. In ONS1 and ONS2, two surface samples were collected, one before (in the morning after cleaning) and one after the administration of 5-FU to the patients. These two rooms, designed for the therapy of the patients, and their respective toilets, were cleaned daily with strong household products by the regular cleaning staff of the hospital. All samples named ‘pre’ are taken after a rigorous standardized cleaning procedure, while the ‘post’ samples are taken directly after the preparation (PD) or administration (ONS) of the 5-FU.

#### 2.7.2. PPEs and Dermal Sampling

In addition to the surface samples, the potential dermal exposure of pharmacy personnel was evaluated by collecting wipe samples from protective gloves that were worn, along with wipe samples taken from the bare hand of the pharmacy personnel. The following nine workers of the hospital were monitored: six pharmacy technicians involved in the preparation of the 5-FU infusion bags (working at PD zone 1 and PD zone 2), and three pharmacists involved in the back-up control of the prepared infusion bags (working at PD zone 3). In order to avoid dermal exposure to cytostatic drugs, the pharmacy technicians preparing the infusion bags wore two pairs of gloves: a first pair of nitrile gloves, with a pair of sterile natural latex gloves on top. The three pharmacists involved in the back-up wore a single pair of nitrile gloves.

#### 2.7.3. Sampling Strategy

When surface sampling was performed, the site area was measured. Yet, this was not always possible. The sampling kit consisted of a 20 mL glass vial containing 1 mL of a mixture of MeOH/H_2_O (80/20) and one, or two, sampling swabs for each surface. After the swab was moistened with the sampling solution, the excess solvent was removed and the surface sampled. For the easily accessible surfaces, a template with a 10 cm × 10 cm opening was used. The first side of the first swab was swiped horizontally over the template opening, then the swab was flipped over and the second side was swiped vertically over the same surface. This swab was then deposited into an empty collection vial. If a second swab was needed (for a surface bigger than 100 cm^2^), the first side of the second swab was swiped diagonally upwards 20 times, then flipped over and the second side swiped diagonally downwards 20 times. The second swab was then deposited into the same collection vial. The samples were extracted the same day as the sample collection, and the resulting solution was stored in the fridge and analyzed within one week.

To evaluate the dermal exposure of the pharmacy technicians preparing the 5-FU infusion bags, we swabbed the surface of the bare hand, before their shift, during their break and after the working shift, along with the outer surface of the gloves that were used for the preparation of the infusion bags. The sampling swabs were moistened with an ethanol/water solution (80/20).

A standardized wiping technique was used to collect dermal exposure samples from the hands and gloves. Starting from the right hand, the palm was wiped five times, from the top of the hand to the start of the fingers and five times across, and, after flipping the swab, the procedure was repeated to sample the back of the hand. A second swab was used for the left hand. The swabs were deposited into collection vials. The samples were extracted the same day as the sample collection, and the resulting solutions were stored in a refrigerator (4 °C) and analyzed within one week.

Since the procedure to remove the gloves has to be strictly followed by the personnel for safety reasons, the gloves have been sampled after they were taken from the hands and wiped in the same manner as the hands.

## 3. Results

### 3.1. UPLC-MS/MS Method

We investigated two different types of ionization sources, to determine the most sensitive one: the more classical electrospray ionization source (ESI) and the UniSpray ionization source (USI). The selection of the preferred source was carried out by evaluating the calibration curve of 5-FU and comparing parameters such as LOD, LOQ, linearity (R^2^) and S/N ratio. Table 2 shows the results of both sources. We found that the USI source was the most sensitive and accurate ionization source for 5-FU quantification on the sampling swabs used in this study. The LOQ obtained from the USI was seven times lower compared to the ESI. The linearity of the calibration curve was slightly better for USI (R^2^: 0.9969) than ESI (R^2^: 0.9937), while the S/N ratio for a swab prepared spiking 1 ng of 5-FU was higher for USI (149.2) compared to the ESI (100.2) source.

### 3.2. Results of the Method Validation

The following step in the validation of the method was the analysis of linearity of the calibration curves by analyzing freshly spiked swabs (n = 3, concentration range from 0.05 to 100 ng/swab of 5-FU) using the USI source. The equations obtained by each of the three experiments were as follows: y = 0.103x, y = 0.118x, y = 0.113x, with all having a coefficient of determination (R^2^) ≥ 0.996.

To determine the precision, four concentrations of 5-FU were spiked, namely 0.3, 1, 10 and 100 ng/swab. The intra-day precision (RSD%) was 9.8%, 3.7%, 4.7%, 2.7%, respectively. The between-day precision (RSD%) was found to be 9.8%, 7.8%, 4.3%, 7.0%, respectively.

The accuracy, expressed as the ratio of calculated concentration to measured concentration, was in the range between 92% and 109%. The LOD of our method corresponds to 0.05 ng/swab, as determined using a signal-to-noise approach (S/N of 2:1). The LOQ obtained for 5-FU was calculated considering the lowest concentration that could be quantified with good accuracy (relative standard error ± 20%), and a precision <10% and a S/N higher than 10:1, corresponding to 0.1 ng/swab. For the environmental sampling, we swabbed 100 cm^2^, so the LOQ recalculated in cm^2^ is 1 pg/cm^2^.

The recovery of 5-FU from sampling swabs was determined for five concentrations (n = 5): 0.5, 1, 10, 50, 100 ng/swab and was found to be 89%, 82%, 99%, 98%, 100%, respectively.

### 3.3. The Evaluation of Environmental and Dermal Exposure in a Public Hospital

#### 3.3.1. Environmental Exposure

A total number of 52 surface samples were collected from a public hospital in Belgium in the following two main designated areas: the pharmacy department (PD) preparing the 5-FU infusion bags, and the outpatient nursing station (ONS) where patients received the 5-FU treatments.

The following twenty-eight samples were collected at the pharmacy department: 14 samples were collected from the preparation surfaces in the laminar flow cabinets (LFC) of PD zones 1 or 2 and 14 samples were collected at PD zone 3. In zones 1 and 2, a pre and post swab was taken five times from the work surface in the LFC when 5-FU infusion bags were prepared; one pre and post swab was taken from the air intake grid of the LFC, and one sample was taken from the floor beneath the LFC on the location of the pharmacy technician preparing the infusion bag. Four samples were collected pre and post the preparation of the 5-FU infusion bags from frequently used devices and objects located at PD zone 2 and 3, such as the touchscreen of a pump, a PC mouse, a door handle, and a calculator. Furthermore, six swab samples were collected to determine 5-FU contamination on specific surfaces in the PD 3, such as the outside of the pack of the infusion bag, the flask containing 5-FU (two measurements on separate days), the container for transport of infusion bags, the contact point on the clave vial adaptor, and the box to collect infusion bags.

**Figure 2:**
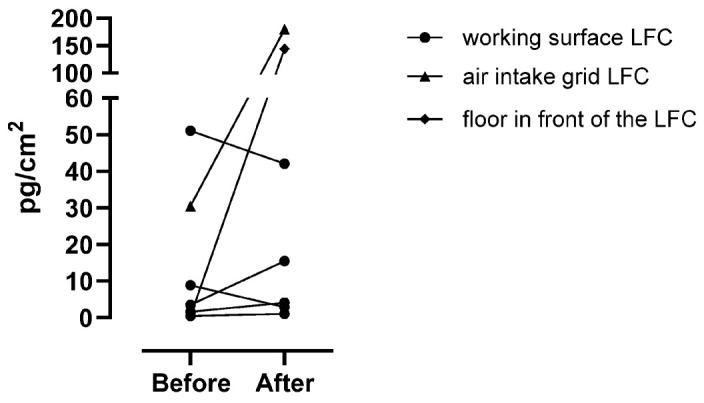
The concentrations of 5-FU on the sampling swabs collected on the working surface of the laminar flow cabinets (LFC) (n = 5), on the air intake grid of the LFC (n = 1), and on the floor in front of the LFC (n = 1) pre and post the preparation of 5-FU infusion bags. The results are expressed in pg/cm^2^ by dividing the amount found on the swab by the sampled surface area (cm^2^).

Figure 2 shows the pre and post surface swabs of the preparation surface in the LFC (n = 5), the air intake grid of the LFC (n = 1), and the floor in front of the LFC where the pharmacy technician prepared the infusion bags (n = 1). Before the preparation of 5-FU infusion bags, 5-FU was found in 4 of the 6 surface swabs, ranging from 1.6 pg/cm^2^ to 51.1 pg/cm^2^. The two swabs that were below the LOQ were given a value of ½ the LOQ, being 0.5 pg/cm^2^. After the preparation of 5-FU infusion bags, the concentration found on the surfaces of the LFC ranged from 1.1 pg/cm^2^ to 180 pg/cm^2^.

**Figure 3:**
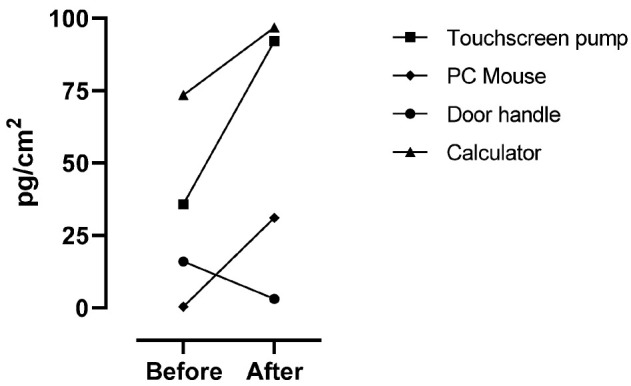
The concentrations of 5-FU on the sampling swabs collected from frequently used devices located in PD zones 2 and 3, pre and post the preparation of 5-FU infusion bags. The results are expressed in pg/cm^2^ by dividing the amount found on the swab by the surface area.

Figure 3 shows the results of surface contamination, before and after the 5-FU manipulation, for the frequently used devices located in PD zones 2 and 3 (see Figure 1). The analysis of the samples collected before the preparation shows a concentration range of 0.5 (½ of the LOQ) to 73.5 pg/cm^2^. The samples collected on the same surfaces after the 5-FU preparation ranged from 3.1 to 96.8 pg/cm^2^.

Furthermore, six single swab samples were collected to determine 5-FU contamination on specific surfaces that could represent potential sources of exposure. We found 168.8 pg/cm^2^ on the outside of the pack of the infusion bag, 121.9 and 346.9 pg/cm^2^ on two vials containing 5-FU, 3.6 pg/cm^2^ on the container for transport of infusion bags, 177,800 pg/cm^2^ on the contact point on the clave vial adaptor, and 86 pg/cm^2^ on the box for the infusion bags collection.

**Figure 4:**
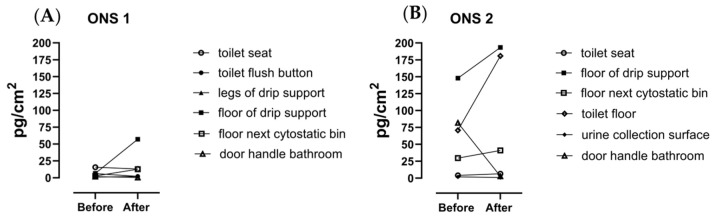
The concentration of 5-FU on the sampling swabs collected on different surfaces at the outpatient nursing stations, ONS1 (**A**) and ONS2 (**B**), before and after the administration of 5-FU infusion bags to patients. The results are expressed in pg/cm^2^ by dividing the amount found on the swab by the surface area.

Two outpatient nursing stations, ONS1 and ONS2, were sampled to evaluate the 5-FU surface contamination. The results are presented in Figure 4. In ONS1, six swabs were collected before and after the treatment on six different surfaces. All swab samples collected before the administration of 5-FU in ONS1 contained 5-FU ranging from 1 to 15 pg/cm^2^. After the administration of 5-FU infusion bags to the patients, the concentration of 5-FU found on the same surfaces ranged from 1.9 to 57.1 pg/cm^2^. The highest surface contamination after the administration of 5-FU was found on the floor of the drip support (Figure 4A).

On the surfaces of ONS2, all 12 swab samples were above the LOQ for 5-FU. Before the administration of the 5-FU infusion bags, the lowest concentration was found at ‘urine collection surface’ (2 pg/cm^2^), while the highest contamination was found at the ‘floor of drip support’ showing a concentration of 148 pg/cm^2^. After the administration of 5-FU, 4 out of 6 swabs showed a higher concentration, ranging from 1.1 to 193 pg/cm^2^. Both the surface contamination on the floor of the drip support, as on the toilets floor, increased substantially after the administration of 5-FU, while the concentration on the door handle of the bathroom decreased 15-fold (Figure 4B).

#### 3.3.2. Protective Gloves and Actual Dermal Exposure

**Figure 5:**
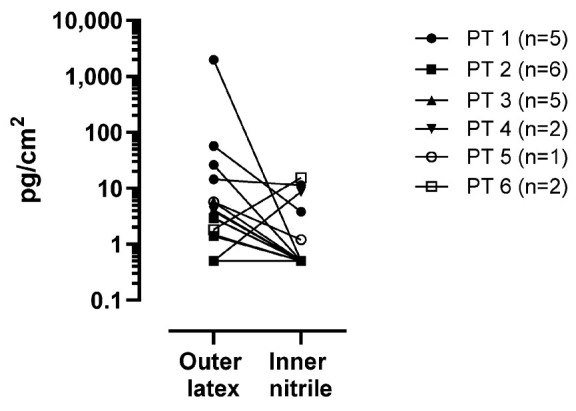
The concentration of 5-FU on the outer latex gloves and inner nitrile gloves worn by pharmacy technicians (PTs) during the preparation of 5-FU infusion bags. For better visualization, the results are presented as pg/cm^2^ in logarithmic scale. Each PT involved in the sampling has their own symbol. The number of gloves analyzed differs between the PTs, due to differences in tasks (n = 1 to 6).

Six pharmacy technicians (PTs), involved in the preparation on 5-FU infusion bags, wore two pair of gloves: an outer natural latex glove and an inner nitrile glove. In total, 42 samples were collected for these workers, divided as follows: 21 for the outer latex gloves and 21 for the internal nitrile gloves; PTs n°1 to n°5 were sampled, respectively, 5, 6, 5, 2 and 1 individual times. PT n°6 was sampled two individual times. The results of 5-FU contamination for the gloves are shown in Figure 5.

On the outer latex gloves 8 out of 21 samples were below the LOQ, which is 0.1 pg/cm^2^. To visualize the higher and lower contamination on the gloves in Figure 5, we opted to give a value of ½ of the LOQ to these samples (0.5 pg/cm^2^). The concentration found on the 13 other latex gloves ranged from 3.9 pg/cm^2^ to 1979 pg/cm^2^. On the nitrile gloves worn underneath the latex gloves, 16 out of 21 samples were below the LOQ. The other 5 gloves showed 5-FU concentrations ranging from 12 pg/cm^2^ to 15.4 pg/cm^2^. In 10/21 occasions, the concentration of 5-FU on the latex gloves was lower on the inner nitrile gloves compared to the outer latex gloves. In 2/21 occasions, we found more 5-FU on the inner nitrile gloves (one from PT 4 and one from PT 7). PT 1 had one occasion on which the concentration of 5-FU was almost similar on the latex glove, compared to the nitrile glove. In 7/21 occasions, we could not find any 5-FU on both gloves (results below LOQ).

In addition to to the collection and analysis of the gloves, we also assessed dermal exposure on the inside and outside of the bare hands of nine PTs using moistened ethanol/water swabs before and after the working shift, and during a break when applicable. In total, twenty-four samples were collected from the hands of these workers. We only found 5-FU on three of the twenty-four hand wipes, as follows: (1) we found 0.43 ng/2 hands 5-FU on the hands of one PT at the beginning of the shift, (2) one PT had 0.95 ng/2 hands 5-FU on their hands at mid-day, (3) while another one had 0.48 ng/2 hands 5-FU on their hands at the end of the shift. Twenty-one samples were below the LOQ 0.4 ng/2 hands, surface of the hands is approximately 800 cm^2^).

## 4. Discussion

In the present study, we aimed to implement a method that was able to detect and quantify very low levels of 5-FU contamination, while still being a fast and relatively simple analytical procedure. Secondly, we tested this method by investigating the surface contamination and dermal exposure of pharmacy personnel to 5-FU in a public university hospital in Belgium. Our results show that our methodology was sensitive enough to quantify extremely low concentrations (pg/cm^2^ range) of 5-FU on surfaces of the hospital pharmacy and two outpatients nursing stations, on the protective gloves of pharmacists, and even on their skin.

To reach the very low LOQ, we compared the following two ionization sources, which initiate the ionization before the MS analysis can occur: the more classical electrospray ionization source (ESI) and the new UniSpray ionization (USI) source. In both sources, the liquid is nebulized via a high-speed nitrogen gas flow. An important difference is that in the USI source, the source is formed by directing a high-velocity nebulized jet from a grounded sprayer onto a cylindrical metal target that is held at a high voltage, located between the sprayer and the ion inlet orifice of the mass spectrometer. This creates a Coandă effect, allowing us to optimize the deflection of the spray from the nebulizer jet towards the ion inlet. When the nebulizer jet impact point is adjusted from a central to an off-axis position on the upper right-hand quadrant of the Ø1.6 mm target, the ion signal intensity rapidly increases until an optimum is reached. This characteristic is highly compound dependent. For 5-FU, we were able to find an optimum, allowing us to increase the sensitivity.

In terms of LOD, LOQ, S/N ratio, and the linearity of the calibration curve, the results of the ESI/USI comparison showed that the USI source is the most sensitive and accurate detection technique for the quantification of 5-FU on sampling swabs.

Instead of the most commonly used wipes [6,23,24,25,26], we selected the TX 714K swabs (Texwipe). These swabs contain less than 0.001 ppb of organic components (according to the manufacturer’s information), allowing us to reach a very low LOQ. Moreover, the swabs have a stick, allowing the operator to collect samples without directly touching the contaminated surfaces or contaminated wipes. A possible limitation of the swabs is the limited surface of the sampling material, which does not allow for the sampling of bigger surfaces due to the possible saturation of the material. We did not evaluate the saturation of the swabs with 5-FU due to safety measures for ourselves, but to avoid possible saturation, and surfaces bigger than 100 cm^2^ were sampled using two different swabs. Due to the high polarity of 5-FU, for environmental samples, the swabs were moistened with MeOH/H_2_O (8/2) for surface sampling and with EtOH/H_2_O (8/2) for skin sampling. Due to the large variety of surfaces investigated, we did not test the actual recovery rates from the different surfaces in the present study. We were able to optimize the method, to reach a LOQ of 100 pg/swab, which corresponds to an effective concentration of 300 pg/mL. Considering a standard sampled surface of 100 cm^2^, the present method can accurately quantify 1 pg/cm^2^ of 5-FU on surfaces and skin.

To validate the sensitivity of our method, we tried to identify surface contamination and dermal exposure to 5-FU in a public hospital, which has strict SOPs in place to handle (dangerous) compounds. Despite these rigorous rules, we were able to retrieve 5-FU in and around the laminar flow cabinets at the pharmacy department, in the median range of 3.5 to 15.5 pg/cm^2^, mostly after the preparation of 5-FU infusion bags. These results show that we are able to detect very low concentrations of 5-FU. Compared to other studies on similar surfaces, the contamination proved to be very low [21,27,28,29,30,31]. Schierl et al. (2009) reported a similar median concentration of 5-FU of 10 pg/cm^2^ for the same surfaces, with the highest concentration ranging up to 20,588 pg/cm^2^ [28].

In multiple studies, it was reported that 5-FU contamination was the highest on the primary packaging of 5-FU or vials. These results suggest that 5-FU contamination may have happened prior to arrival at the site where it will be used, thereby demonstrating that personnel should be aware of possible contamination on the primary packaging of cytostatic drug bags or vials, and standard decontamination procedures should be considered during manufacturing [15,26,32,33,34].

We also collected swab samples from items that are often used and touched, such as the touchscreen of a pump, the PC mouse, the door handle, and the calculator. All these items contain a higher concentration of 5-FU after the preparation of the infusion bags. Yet, the door handle was less contaminated by 5-FU after the preparation, probably because the door handle is one of the most touched items in the room, and the amount on this surface decreases over time, representing certainly a potential source for dermal exposure.

In addition to the pharmacy department, we have also collected surface samples from two outpatient nursing stations (ONS1 and ONS2) where patients in a day-by-day routine receive the 5-FU therapy, can recuperate, and go home again. Also, in these areas, we could detect 5-FU, but the concentrations were even lower than in the pharmacy department, ranging from 0.001 to 0.193 ng/cm^2^. Overall, we could find more 5-FU after the treatment of the patients, except for the door handle of the bathroom and the toilet seat. On the floor around the toilet of ONS2, we found relatively high concentrations of 5-FU even before the treatment, which further increased after the patients left the room. This type of contamination was previously already indicated as possibly the most contaminated area, due to the urinary excretion of an unmodified cytostatic drug [4,32,33]. We found similar concentrations around the toilet, as previously described by other authors [4,31,33,34]. However, it remains difficult to determine whether this contamination is the result of urine droplets or of cross-contamination via protective clothes, or whether it occurs during cleaning tasks. Unlike the preparation area of the pharmacy department, where rigorous cleaning procedures are performed by pharmacy personnel using strong decontaminating products, the cleaning procedure in the ONS is performed by professional cleaning personnel using general strong cleaning products which are not specifically made for these hazardous compounds. It would have been very interesting if we could have included the cleaning staff to determine dermal exposure to 5-FU, but due to practical reasons this was not feasible.

Currently, there are no official surface contamination limits for cytostatic drugs. Therefore, we refer to the Monitoring-Effect Study of Wipe Sampling in Pharmacies (MEWIP) conducted in Germany [29]. They proposed a limit value, substance-independent, of 0.1 ng/cm^2^ based on the 90th percentile of the compound found in the highest concentrations in MEWIP. The authors of this study also suggest that for concentrations lower than 0.1 ng/cm^2^, the environment should be monitored at least once a year. If the surfaces have contamination levels between 0.1 and 10 ng/cm^2^, a surface assessment should be performed within three to six months and actions should be taken if necessary. When the surface contamination with cytostatic drugs exceeds 10 ng/cm^2^, immediate action is required. In the pharmacy department of our study, only two samples, out of fourteen collected on the surfaces of the laminar flow cabinets, were above 0.1 ng/cm^2^. Both these samples were collected after the preparation of infusion bags, implying that the cleaning procedure for these surfaces is more than efficient enough. Apart from the samples collected on the outside of the vials and infusion bags, only three samples from the outpatient nursing station showed a 5-FU concentration above 0.1 ng/cm^2^. These were samples collected on the floor of the drip support and on the toilet floor.

In addition to surface sampling, we also evaluated whether we were able to assess the glove contamination of 5-FU. The use of nitrile gloves in the pharmacy cytotoxic workroom is mandatory and the gloves must be replaced at regular intervals [33]. In our study, pharmacy personnel working in the preparation zone strictly follow the guidelines and even add an extra latex glove on top of the nitrile gloves. These gloves are changed at least every 30 min. We found more 5-FU on the outer latex gloves most of the time, compared to the surface of the inner nitrile gloves. Yet, we did find 5-FU on the inner nitrile gloves. With the current study set-up, we cannot explain whether the 5-FU on the inner nitrile gloves comes from cross-contamination during the removal of the outer latex gloves, or that the actual permeation of 5-FU through outer latex gloves onto the inner nitrile occurred. On the bare hands of the personnel, only three samples showed a concentration of 5-FU above the LOQ, with extremely low concentrations of 5-FU.

The exclusive evaluation of 5-FU exposure in our study represents a limitation of the study. The personnel in the pharmacy department come into contact with many other compounds, including other cytostatic drugs. Co-exposure to other cytostatic drugs could certainly occur. Yet, since 5-FU is generally one of the most investigated compounds in other studies, and considering the large amount of 5-FU used in this hospital, it could be considered a valid proxy for assessing the overall exposure to cytostatic drugs among hospital personnel and a good surrogate to evaluate the safe manipulation and the cleaning procedure [7,27,35,36,37]. Another limitation is the extrapolation of the limited surface that is sampled (100 cm^2^) versus the sometimes much larger surface it reflects, e.g., the floor or the surface of a laminar flow cabinet.

In conclusion, our simple, fast UPLC-MS/MS method with USI was able to detect very low concentrations of 5-FU contamination in an environment handling cytostatic agents on a daily basis. Considering the large quantities of 5-FU that are used, we can conclude that the current SOPs on manipulation, preparation, and cleaning, along with the protective gloves that are in place, can be considered very adequate.

## Figures and Tables

**Table 1:** The parameters of the electrospray (ESI) and UniSpray (USI) ionization sources.

Ionization Source	ESI	USI
Source Temperature (°C)	150	150
Desolvation Temperature (°C)	450	450
ESI Capillary Voltage (Kv)/USI Impactor Voltage (Kv)	0.5	1
Cone Gas Flow (L/H)	20	50
Desolvation Gas Flow (L/H)	1000	1000

**Table 2:** A comparison between the electrospray ionization source (ESI) and the UniSpray ionization source (USI) obtained after the injection of a calibration curve.

	ESI	USI
Mass Transition		
LOD	0.3 ng/swab	0.05 ng/swab
LOQ	0.7 ng/swab	0.1 ng/swab
R^2^	0.9937	0.9969
Slope	y = 0.103x	y = 0.110x
S/N Ratio (1 ng/swab)	100.2	149.2

Range: 0.05–100 ng/swab (n = 3), LOD = limit of detection, LOQ = limit of quantification, R^2^ = coefficient of determination, S/N = signal-to-noise ratio.

## Data Availability

All numerical data presented in this study is available on request to the corresponding author.
